# Anti-Atherosclerotic Effect of *Hibiscus* Leaf Polyphenols against Tumor Necrosis Factor-alpha-Induced Abnormal Vascular Smooth Muscle Cell Migration and Proliferation

**DOI:** 10.3390/antiox8120620

**Published:** 2019-12-05

**Authors:** Cheng-Chung Chou, Chi-Ping Wang, Jing-Hsien Chen, Hui-Hsuan Lin

**Affiliations:** 1 Laboratory Medicine, Antai Medical Care Corporation Antai Tian-Sheng Memorial Hospital, Pingtung County 928, Taiwan; s109017@yahoo.com.tw; 2 Department of Clinical Laboratory, Chung Shan Medical University Hospital, Taichung City 0201, Taiwan; cshb015@csh.org.tw; 3 Department of Nutrition, Chung Shan Medical University, Taichung City 40201, Taiwan; 4 Department of Medical Laboratory and Biotechnology, Chung Shan Medical University, Taichung City 40201, Taiwan

**Keywords:** proliferation, migration, vascular smooth muscle cells, atherosclerosis, tumor necrosis factor-alpha, *Hibiscus* leaf polyphenols

## Abstract

The proliferation and migration of vascular smooth muscle cells (VSMCs) are major events in the development of atherosclerosis following stimulation with proinflammatory cytokines, especially tumor necrosis factor-alpha (TNF-α). Plant-derived polyphenols have attracted considerable attention in the prevention of atherosclerosis. *Hibiscus* leaf has been showed to inhibit endothelial cell oxidative injury, low-density lipoprotein oxidation, and foam cell formation. In this study, we examined the anti-atherosclerotic effect of *Hibiscus* leaf polyphenols (HLPs) against abnormal VSMC migration and proliferation in vitro and in vivo. Firstly, VSMC A7r5 cells pretreated with TNF-α were demonstrated to trigger abnormal proliferation and affect matrix metalloproteinase (MMP) activities. Non-cytotoxic doses of HLPs abolished the TNF-α-induced MMP-9 expression and cell migration via inhibiting the protein kinase PKB (also known as Akt)/activator protein-1 (AP-1) pathway. On the other hand, HLP-mediated cell cycle G0/G1 arrest might be exerted by inducing the expressions of p53 and its downstream factors that, in turn, suppress cyclin E/cdk2 activity, preventing retinoblastoma (Rb) phosphorylation and the subsequent dissociation of Rb/E2F complex. HLPs also attenuated reactive oxygen species (ROS) production against TNF-α stimulation. In vivo, HLPs improved atherosclerotic lesions, and abnormal VSMC migration and proliferation. Our data present the first evidence of HLPs as an inhibitor of VSMC dysfunction, and provide a new mechanism for its anti-atherosclerotic activity.

## 1. Introduction

Atherosclerosis is considered a chronic inflammatory process and involves a complex pathophysiological effect, including endothelial dysfunction, low-density lipoprotein (LDL) oxidation, foam cell formation, and vascular smooth muscle cell (VSMC) proliferation and migration at different stages of this disease [1,2]. Elevated plasma LDL concentration contributes to the initiation of atherosclerosis [3]. Oxidized LDL triggers endothelial cells to release chemokines in contribution to recruitment of monocytes, resulting in the transformation of the lipid-laden macrophages into foam cells [3]. In the lesion progression, these activated macrophages still secrete proinflammatory cytokines, especially tumor necrosis factor-alpha (TNF-α), which enhances VSMC migration and proliferation [1,3]. Subsequently, VSMC transforms and proliferates into foam cells, and thus the accumulation of foam cells leading to fatty streaks results in the formation of atherosclerotic plaques [2]. Thus, inhibition of abnormal VSMC migration and proliferation is an attractive strategy for clinical therapy of atherosclerosis and restenosis after percutaneous coronary interventions.

VSMC is normally quiescent, but upon vascular injury, it transforms into a more synthetic phenotype with progressively increasing capacity for activation, proliferation, and migration [1,4]. In the atherosclerotic process, VSMC migrates from the media to the intima, forms the neointima progressively with abundant levels of extracellular matrix (ECM) proteins, and then eventually leads to plaque formation [2]. Identification of key proteins involved in the process, such as matrix metalloproteinases (MMPs), is vital for understanding atherosclerosis and devising new therapies. MMPs are a subfamily of the metzincin superfamily of endogenous proteinases that break down components of ECM. Among them, the gelatinases (MMP-2 and MMP-9) degrade efficiently native collagen types IV and laminin, and promote a VSMC migratory phenotype [5]. Moreover, the gene expression of MMPs is majorly regulated by the transcriptional factors, such as activator protein-1 (AP-1) or nuclear factor-kappaB (NF-κB) through the serine/threonine protein kinase PKB (also known as Akt) or extracellular signal-regulated kinase (ERK) pathways, or by the MMP protein activators or inhibitors. One review study concluded that oxidative stress could enhance MMP activity and expression [6], and recent studies further indicate that MMP-mediated ECM remodeling is modulated by reactive oxygen species (ROS) [7]. Hence, MMPs and their regulatory signaling have been considered as promising targets for anti-atherosclerotic agents [8].

In arterial media, VSMC is at low proliferative indices (<0.05%) and remains in the G0/G1 phase of the cell cycle [4]. However, VSMC re-enters into the cell cycle from the quiescent state to proliferate under the stimulation of several cytokines in pathological processes, which plays an important role in the development of atherosclerosis [1]. VSMC begins to divide in response to cytokines, exits the G1 phase, and then enters the S phase. During the G1/S transition, cyclin D1/cyclin-dependent kinase (cdk) 4 and cyclin E/cdk2 complexes are required. The complexes participate in the hyperphosphorylation of retinoblastoma (Rb) tumor suppressor, leading phosphorylated Rb (p-Rb) to release E2F transcription factor, allowing the cells to progress into S phase [9]. The kinase activities of these cyclin/cdk complexes are regulated by cdk inhibitors (cki), including p16, p21, and p27. The gatekeeper of the mammalian cell cycle, p53, plays a key role in controlling G0/G1 arrest through its downstream factor, such as p21 [10].

Previous studies have reported that *Hibiscus* leaf, an edible part of *H. sabdariffa* Linne (*Malvaceae*) [11], possesses hypoglycemic [12], hypolipidemic [13,14], and antioxidant [13,15] effects, as demonstrated by various experimental models (Table S1). For the standardization of each extract, our studies also indicated that (–)-epicatechin gallate (ECG; 16.5 ± 5.6%) was identified to be present in the highest level in *Hibiscus* leaf polyphenols (HLPs), followed by ellagic acid (EA; 10.31 ± 3.43%) and catechin (Cat; 7.4 ± 2.6%), and traces of only quercetin (Que; 0.8 ± 0.4%) and ferulic acid (FA; 0.7 ± 0.3%) were detected (Table S2) [14]. In this regard, the aqueous and methanol extracts of *H. sabdariffa* leaves showed anti-atherogenic effects in hyperlipidemia animals induced by cholesterol [11,12], and inhibited foam cell formation, as well as protected endothelial cells from injury in vitro [11,16]. Our recent studies also revealed that *Hibiscus* leaf aqueous extract, due to its high content in polyphenols, has apoptotic and anti-migratory effects on prostate cancer cells [17,18]. However, little information is available on the isolation and characterization of a polyphenolic extract of *H. sabdariffa* leaves. In the present study, HLP was partially characterized by biochemical and spectroscopic assays, and evaluated for the ability to inhibit TNF-α-stimulated VSMC dysfunction.

Many studies have indicated that plant-derived polyphenols have various pharmacological and biological effects, such as antioxidant, anti-inflammatory, anti-hyperlipidemia, anti-diabetes, anti-atherogenic, and anti-tumor abilities [19]. Furthermore, although the protective effects of HLPs on endothelial cells and macrophages have been demonstrated previously, the in vivo function and the molecular target of HLPs on VSMC remain to be elucidated in cardiovascular microenvironment. Using a model of VSMC exposed to TNF-α and the well-established atherosclerotic rabbit experiment, to our knowledge, this is the first report revealing the TNF-α-antagonist potential of HLPs in vitro and in vivo.

## 2. Materials and Methods 

### 2.1. Preparation of HLP and Detection of Polyphenolic Compounds

One hundred grams of *H. sabdariffa* L. (Malvaceae) dried leaves, obtained from Taitung City, Taitung Country, Taiwan, were extracted three times with methanol (300 mL) at 50 °C for 3 h, and the samples were filtered after each extraction. The methanol was evaporated under reduced pressure, and the residue was dissolved in 500 mL of distilled water at 50 °C and extracted with 200 mL of hexane to remove pigments. The aqueous phase was extracted three times with 180 mL of ethyl acetate, and the solvent was removed from the extract with a vacuum rotary evaporator. The residue was re-dissolved in 250 mL of distilled water and was lyophilized to obtain about 2.5 g of HLP. The polyphenolic components of HLP were further analyzed as follows. All reagents and pure compounds were purchased from Sigma-Aldrich Chemical Co. (St. Louis, MO, USA). Total phenolic acid content was determined by the Folin-Ciocalteau method [20] using gallic acid (GA) as a standard. To start, 0.1 mg of HLP was first dissolved in a tube with 1 mL of distilled water, and 0.5 mL of Folin-Ciocalteu reagent (2 N) was added and mixed thoroughly. After 3 min, 3 mL of Na_2_CO_3_ solution (2%) was added, and the mixture was allowed to stand for 15 min. The absorbance of the mixture at 750 nm was measured on a spectrophotometer (Beckman Coulter DU 730, Brea, CA, USA). The concentration of total flavonoid was assayed according to the Jia method [21]. A standard curve using rutin (Rut) was also prepared. Next, 0.5 mL of HLP (1 mg/mL) was diluted with 1.25 mL of distilled water. Afterwards, 75 μL of NaNO_2_ solution (5%) was added to the mixture. After an interval of 6 min, 150 μL of AlCl_3_·6H_2_O solution (10%) was added, and the mixture was allowed to stand for another 5 min. Then, 0.5 mL of NaOH (1 M) and 2.5 mL of distilled water were added. The solution was mixed, and the absorbance was immediately measured against the prepared control at 510 nm. The polyphenolic components of HLP were confirmed by high performance liquid chromatography (HPLC) system using a Hewlett-Packard Vectra 436/33 N system with a diode array detector (all from Waters Corp., Milford, MA, USA). The HLP was filtered through a 0.45 μm filter disc, and then 20 μL of HLP was injected onto a 5 μm RP-18 column (4.6 × 150 mm i.d.; Phenomenex, Inc., Torrance, CA, USA). The mobile phase contained two solvents, including solvent A (formic acid/water = 10:90) and solvent B (formic acid/acetonitrile/water = 10:30:60), run by a linear gradient method at room temperature as follows: From 10% solvent B to 40% solvent B (flow rate = 1.0 mL/min) over 25 min. The chromatography was monitored at 240 and 345 nm, and an ultraviolet (UV) spectrum (Beckman Coulter Inc., Brea, CA, USA) was collected to confirm peak purity. The HPLC analysis of 10 kinds of standard polyphenols showed the retention times (RT) as follows: GA (4.58 min), protocatechuic acid (PCA, 7.50 min), Cat (9.39 min), ECG (11.21 min), EA (13.29 min), Rut (14.01 min), ρ-coumaric acid (CA, 14.44 min), FA (15.28 min), Que (21.57 min), and naringenin (Nar, 24.48 min), respectively. Consistent with our previous study [16], the yield of HLP was approximately 25.0%, and polyphenols were indeed present in HLP (Table S2).

### 2.2. Cell Culture

A rat VSMC cell line A7r5 was purchased from the Bioresource Collection and Research Center. A7r5 cells were cultured in Dulbecco’s modified Eagle’s medium (DMEM) supplemented with 10% fetal bovine serum, 1% penicillin-streptomycin mixed antibiotics, 1% glutamine, and 1.5 g/L sodium bicarbonate (all regents from Hyclone, Logan, Utah, USA). All cell cultures were maintained at 37 °C under 95% moisturized air with 5% CO_2_. Before cell treatments, A7r5 cells were seeded onto each 60 mm Petri dish (Corning Inc, Corning, NY, USA) at a density of 10^5^ for 24 h. For induction of VSMC dysfunction, A7r5 cells at 70% confluence were serum-starved for 24 h and treated with TNF-α (10 ng/mL; Sigma-Aldrich, St Louis, MO, USA) for 24 h.

### 2.3. 3-(4,5-Dimethylthiazol-Zyl)-2,5-Diphenyltetrazolium Bromide (MTT) Assay

A7r5 cells were seeded onto each 24-well plate (Corning Inc, Corning, NY, USA) at a density of 10^5^ cells/mL, and treated with various concentrations of TNF-α (0–20 ng/mL) alone or TNF-α (10 ng/mL) in combination with HLP (0–1.0 mg/mL) for 24 or 48 h. Thereafter, the medium was changed, MTT (0.1 mg/mL, Sigma, St. Louis, MO, USA) was added for next 4-h incubation. The viable cell number was directly proportional to the formazan production, which was solubilized in isopropanol and detected at 563 nm with a spectrophotometer. The MTT assay was used to evaluate the effect of TNF-α alone or TNF-α and/or HLP on cell viability, and to determine the non-cytotoxic doses of HLP, as described by Chen et al. [16].

### 2.4. Gelatin Zymography Protease Assay

The activities of MMP-2 and MMP-9 in the serum-free conditioned medium were evaluated by gelatin zymography according to a previously described method by Huang et al. [22]. In short, samples were prepared with standard sodium dodecyl sulfate (SDS)-gel loading buffer containing 0.01% SDS (Sigma-Aldrich, St Louis, MO, USA). The prepared samples (25 μg total protein) were not boiled before loading, but subjected to electrophoresis on 8% SDS polyacrylamide gels (1.0-mm-thick, acrylamide/bis-acrylamide = 30/1.2) containing 0.1% gelatin (Sigma-Aldrich, St Louis, MO, USA). After electrophoresis, the gel was washed twice with 100 mL distilled water containing 2% Triton X-100 (Sigma-Aldrich, St Louis, MO, USA) on a shaker for 30 min at room temperature to remove SDS, and incubated in 100 mL reaction buffer (0.02% NaN_3_, 10 mM CaCl_2_ and 40 mM Tris-HCl (pH 8.0)) at 37 °C for 12 h. The gel was further stained with Coomassie brilliant blue R-250 dye (Abcam plc, Cambridge, UK) followed by destaining with methanol/acetic acid/water (50:75:875, *v/v/v*).

### 2.5. Real-Time Reverse Transcription Polymerase Chain Reaction (RT-PCR) 

Total RNAs were extracted using a TRIzol reagent (Invitrogen, Life Technologies, Carlsbad, CA, USA) according to the manufacturer’s instructions, as described by Chiu et al. [18]. In general, the mRNA levels were analyzed by quantitative real-time RT-PCR using a Bio-Rad iCycler system (Bio-Rad, Hercules, CA, USA), and normalized to the housekeeping gene, β-actin. The sequences of primers (MDBio Inc., Taipei, Taiwan) used in the experiments are listed in Table S3.

### 2.6. Protein Isolation and Western Blotting

The preparation of cytosolic and nuclear fractions of the cells was performed using the Nuclear and Cytoplasmic Extraction Reagent Kit (Thermo Scientific, Rockford, IL, USA), described by Chiu et al. [18]. In brief, the harvested cells were washed with phosphate-buffered saline (PBS) and incubated on ice in Reagent A for 2 min. Reagent B was added, and the mixture was further incubated on ice for 5 min. Reagent C was added, and the contents were mixed by inverting the tube several times, followed by centrifugation (700× *g*) at 4 °C for 10 min. The supernatant (cytosol) was collected and centrifuged (12,000× *g*) at 4 °C for 15 min. Then, the nuclear pellet was washed twice with wash buffer (10 mM Tris-HCl (pH 7.5), 0.4% Nonidet P-40, and 10 mM KCl) to remove non-lysed cells. A protease inhibitor cocktail (Bio-Rad Labs., Hercules, CA, USA) was added to all solutions before use. Western blot analysis was carried out according to a previously described method by Chen et al. [16]. Whole cell lysate was prepared using sample buffer containing 2% SDS, 10% glycerol, 5% β-mercaptoethanol, and 50 mM Tris-HCl (pH 6.8), and then extracted using sonication. Equal amounts of proteins were separated by 8–15% SDS–polyacrylamide gels and transferred to nitrocellulose membranes (Bio-Rad Labs., Hercules, CA, USA). In order to block non-specific binding, the nitrocellulose membranes were incubated with 5% nonfat dry milk for 1–2 h at 4 °C, and then overnight with polyclonal first antibodies against MMP-2, MMP-9, p-Akt, Akt, p-ERK, ERK, c-Jun, c-Fos, NF-κB, p-p53, p53, p21, p27, p16, PCNA (proliferating cell nuclear antigen), E2F, and p-Rb were from Santa Cruz Biotech (CA, USA). In the subsequent day, the blots were incubated with the appropriate horseradish peroxidase-conjugated secondary antibodies (goat anti-rabbit IgG or goat anti-mouse IgG), from Sigma-Aldrich (St Louis, MO, USA), for 1 h, and detection was performed using an enhanced chemiluminescence (ECL) reagent (Amersham, Arlington Heights, IL, USA). The cytosolic and nuclear protein were respectively determined by Western blotting using anti-β-actin and anti-C23 antibodies, purchased from Santa Cruz Biotechnology Inc. (Santa Cruz, CA, USA),as loading controls. Protein level was quantified by densitometry using FUJIFILM-Multi Gauge V2.2 software (Fujifilm, Kyoto, Japan).

### 2.7. AP-1 and NF-κB Binding Assay

DNA-binding activities of AP-1 and NF-κB in nuclear extracts were assayed by electrophoretic mobility shift assay (EMSA) with biotin-labeled double-stranded AP-1 or NF-κB oligonucleotides (MDBio Inc., Taipei, Taiwan), as described by Chiu et al. [18]. EMSA was carried out by using the Lightshift kit from Pierce (Rockford, IL, USA). Binding reactions containing 10 μg of nuclear extracts, 1 μg poly (dI·dC), 12.5 μg poly-l-lysine, 2 pmol of oligonucleotide probe, and 2 μL of 10× binding buffer were incubated for 20 min at room temperature. Protein-DNA complexes were separated by electrophoresis on a 6% non-denaturing acrylamide gel, transferred to positively charged nylon membranes (Millipore, Bedford, MA, USA), and then UV cross-linked. Gel shifts were visualized with a streptavidin-horseradish peroxidase followed using chemiluminescent detection.

### 2.8. Wound-Healing Migration Assay

To study the possibility that HLP alter migration of VSMC-treated TNF-α, the cell medium was replaced with serum-containing medium following the treatments of TNF-α (10 ng/mL) in the absence or presence various concentrations (0, 0.01, 0.05, and 0.10 mg/mL) of HLP, and the monolayers were wounded using scraping with a 20-μL pipette tip. At the indicated times (0, 24, and 48 h) after scraping, the above-treated cells were washed twice in PBS (pH 7.4). The cells were photographed using a phase-contrast microscope (Olympus, Tokyo, Japan) [23].

### 2.9. Boyden Chamber Invasion Assay

To test the effect of HLP on the in vitro invasiveness of VSMC-treated TNF-α, a modified Boyden chamber (Neuro Probe, Cabin John, MD, USA) invasion assay coating with a layer of Matrigel (25 mg/50 mL; Sigma-Aldrich, St Louis, MO, USA) was used [23], and was applied to polycarbonate membrane filters with an 8.0 μm pore size (Nucleopore, Pleasanton, CA, USA). Afterwards, the membrane was fixed with methanol, and then stained with 10% Giemsa (Sigma-Aldrich, St Louis, MO, USA). The image of cells invaded through the membrane was capture and counted under the light microscope.

### 2.10. Cell Growth Curve Analysis

VSMC was seeded into a 6-well culture plate at a density of 7 × 10^4^ cells/mL, and then incubated with TNF-α (10 ng/mL) in the absence or presence various concentrations (0, 0.2, and 0.5 mg/mL) of HLP for 24 h. The cell numbers were further counted using the Corning Cell Counter with a reusable glass counting chamber (Corning Inc, Corning, NY, USA) each day for 2 days. On the basis of the mean number of cells in these wells, the growth curves were formed.

### 2.11. Bromodeoxyuridine (BrdU) Cell Proliferation Assay

To identify the cells in S phase of cell cycle, the BrdU cell proliferation assay (Oncogene, Cambridge, MA, USA) was carried out according to the manufacturer’s manual. In brief, A7r5 cells were seeded into a 96-well plate (4 × 10^3^ cells/well) and grown in DMEM medium supplemented with 5% FBS overnight. The cells were rinsed once with serum-free medium, and then treated with TNF-α (10 ng/mL) in the presence or absence of various concentrations (0.2 and 0.5 mg/mL) of HLP in serum-free medium for 24 h. In most of the experiments, pulse labeling of synthesized DNA was used. For this, the BrdU label was added 1 h before the experimental end. The cells were fixed, denatured, and probed with anti-BrdU antibody. Absorbance was determined at dual wavelengths of 450 and 540 nm in a microplate reader system (Bio-Rad Labs., Hercules, CA, USA). Proliferative value (BrdU incorporation) was expressed as a percentage of absorbance of the treated cells to the absorbance of the non-treated control cells. The BrdU incorporation of the control group was set to 100%.

### 2.12. Cell Cycle Analysis by DNA Content

The quantification of cell cycle distribution was examined using a FACScan laser flow cytometer (Becton Dickinson, San Jose, CA, USA). The VSMC was treated with TNF-α (10 ng/mL) in the absence or presence various concentrations (0.2 and 0.5 mg/mL) of HLP for 24 h; collected, rinsed with PBS twice; fixed in 70% ethanol at –20 °C overnight; and then stained with propidium iodide (PI) solution (20 μg/mL of PI, 20 μg/mL of RNase A, and 0.1% Triton X-100; all chemicals from Sigma-Aldrich, St Louis, MO, USA) for 20 min in the dark at room temperature. Each phase of cell cycle was presented as the cell number versus the DNA content as indicated by the intensity of fluorescence, and gated into subG1, G0/G1, S, and G2/M phases with CELLQuest Version 3.3 software (Becton Dickinson, San Jose, CA, USA).

### 2.13. Immunoprecipitation

For detection of protein-protein interaction, immunoprecipitation was carried out. In short, 500 μg of protein from cell lysates was precleared with protein A–agarose beads (Pierce Biotechnology, Rockford, IL, USA), followed by immunoprecipitation using polyclonal antibodies against cdk2 or E2F, purchased from Santa Cruz Biotechnology Inc. (Santa Cruz, CA, USA). Immune complexes were harvested with protein A, and immunoprecipitated proteins were then assayed by Western blotting, as above. Immunodetection was performed using polyclonal anti-cyclin E or anti-Rb antibodies (Santa Cruz Biotechnology Inc., Santa Cruz, CA, USA).

### 2.14. Intracellular ROS Assay

The fluorescent probe, dichlorofluorescin diacetate (DCFH-DA), purchased from Enzo Life Sciences Inc. (Farmingdale, NY, USA), was used to determine the effect of HLP on intracellular ROS generation by TNF-α stimulation. In brief, the confluent A7r5 cells in the 6-well plates at 10^5^ cells/well were treated with TNF-α (10 ng/mL) in the absence or presence various concentrations (0.2 and 0.5 mg/mL) of HLP for 24 h. After removing the treated cells from the wells, the cells were incubated with 2 µM of DCFH-DA at 37 °C for 30 min. The fluorescence intensity of intracellular ROS production was evaluated at an excitation and emission wavelength of 485 and 530 nm, respectively, using Muse™ Cell Analyzer (EMD Millipore Corporation, Merck Life Sciences, KGaA, Darmstadt, Germany). Values were expressed relative to the fluorescence signal of the control.

### 2.15. Evaluation of Atherosclerotic Lesions In Vivo

New Zealand white male rabbits weighing between 1800 and 2200 g were randomly divided into five experimental groups as follows: Group I, normal control group (Purina Lab Diet 5031); group II, high-fat diet (HFD); group III, HFD with 0.5% HLP group (HFD + 0.5% HLP); group IV, HFD with 1% HLP group (HFD + 1% HLP); and group V, normal diet with 1% HLP group (cytotoxicity group of HLP). The rabbits in groups II, III, and IV were fed on a HFD containing 95.7% standard Purina Chow (Purina Mills Inc., Louis, MI, USA), 1.3% cholesterol, and 3% lard oil (Sigma-Aldrich, St Louis, MO, USA) for 25 weeks to induce the atherosclerotic process., In groups III, IV, and V, the rabbits were treated with oral feeding 0.5% or 1% HLP at the same time. The dose regimen for these groups was based on a previous study published by Chiu et al. [18]. For the care and use of laboratory animals, the use of all rabbits was reviewed and approved by Chung Shan Medical University animal care committee according to the guidelines of the Institutional Animal Care and Use Committee (IACUC approval number: 893). After 25 weeks of supplementation, aortic arches from each rabbit were collected and then stained with hematoxylin and eosin (H & E) for the pathological analysis. Serum was also collected and stored at –80 °C until measurements of serum biochemical parameters and TNF-α using a cytoscreen immunoassay kit (BioSource International, Camarillo, CA, USA). For immunohistochemistry (IHC), commercial monoclonal anti-alpha smooth muscle actin (α-SMA, a marker of VSMC migration), obtained from Santa Cruz Biotechnology Inc. (Santa Cruz, CA, USA), and anti- PCNA (a marker of VSMC proliferation) were used for target detection in the paraffin-embedded tissues.

### 2.16. Statistical Analysis

In vitro data are reported as means ± standard deviation (SD) of three independent experiments. The in vivo effect of each treatment was analyzed from 6 rabbits (*n* = 6) in each group. Statistical significances of difference throughout this study were evaluated by one-way analysis of variance (ANOVA). *p* < 0.05 was considered statistically significant.

## 3. Results

### 3.1. Non-Cytotoxic Doses of HLP Inhibits TNF-α-Induced Cell Viability Loss and MMP Activation 

**Figure 1:**
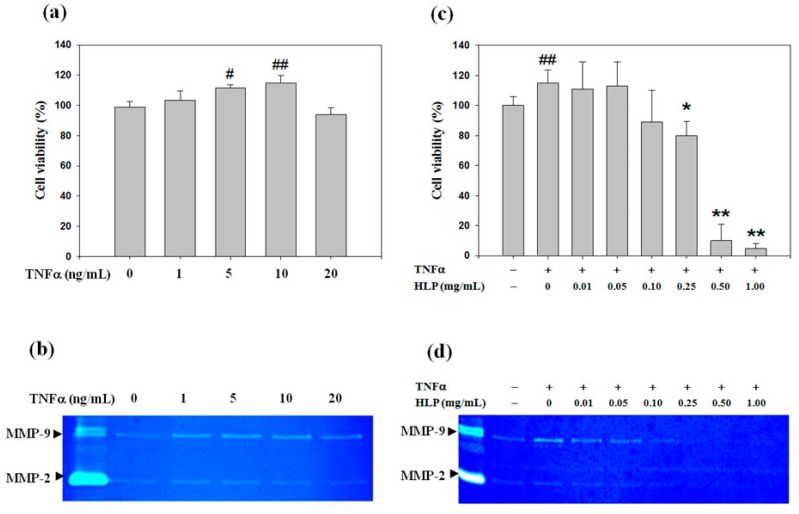
Effect of tumor necrosis factor-alpha (TNF-α) or/and *Hibiscus* leaf polyphenol (HLP) on cell viability and matrix metalloproteinase (MMP) activities in vascular smooth muscle cells (VSMCs). (**a**) A7r5 cells were treated with various concentrations (0–20 ng/mL) of TNF-α for 24 h. Cell viability was analyzed by MTT assay. (**b**) A7r5 cells in serum-free medium were treated with various concentrations of TNF-α for 24 h. The culture medium of cells after treatment was subjected to gelatin zymography to analyze the MMP activity. (**c**) A7r5 cells were treated with TNF-α (10 ng/mL) in the absence or presence of various concentrations (0.01, 0.05, 0.10, 0.25, 0.50 and 1.00 mg/mL) of HLP for 24 h. Cell viability was analyzed by MTT assay. The quantitative data are presented as mean ± standard deviation (SD) (*n* = 3) from three independent experiments. # *p* < 0.05, ## *p* < 0.01 compared with the control. * *p* < 0.05, ** *p* < 0.01 compared with the TNF-α group. (**d**) A7r5 cells in serum-free medium were treated with TNF-α in the absence or presence of various concentrations of HLP for 24 h. The culture medium of cells after treatment was subjected to gelatin zymography to analyze the MMP activity. The result is representative of at least three independent experiments. +, added; −, non-added.

A7r5 cell viability was investigated following incubation with a range of concentrations (from 1 to 20 ng/mL) of TNF-α for 24 h, and it was found that TNF-α at low concentrations (lower than 10 ng/mL) dose-dependently increased the cell viability. However, above the dose of 10 ng/mL, TNF-α reduced about 10% of cell viability (Figure 1a). Because MMPs break down components of ECM, which is a crucial role in the process of VSMC migration [7,8], the effect of TNF-α on MMP activities was then tested by gelatin zymography in serum-free conditioned medium to identify the contribution of MMP-2 or MMP-9 to the pro-migratory ability of TNF-α. As shown in Figure 1b, MMP-9 activity was tremendously increased by TNF-α in a concentration-dependent manner, whereas MMP-2 activity was less affected. According to the results, to provide a maximum dynamic range for quantifying the VSMC proliferative and pro-migratory responses, cell incubation with 10 ng/mL of TNF-α for 24 h was chosen in all subsequent experiments.

In our previous study, HLP at concentrations of > 0.05 mg/mL was demonstrated to be an antioxidant agent, as tested by its 1,1-diphenyl-2-picrylhydrazyl (DPPH) scavenging effect and ability to inhibit LDL oxidation in standard antioxidant evaluation [14], as shown in Table S4. Next, a preliminary screening was performed to study the effect of HLP alone (Figure S1) or together with TNF-α at 10 ng/mL (Figure 1c) on A7r5 cell growth for 24 h, using the MTT assay. The viability of A7r5 was significantly decreased by 0.25, 0.50, and 1.0 mg/mL of HLP in the absence or presence of TNF-α in a dose-dependent manner, when receptively compared to the control and TNF-α alone group. In order to study whether HLP is an inhibitor of cell migration and MMP-9 activation in the TNF-α-treated VSMC, the effect of HLP on A7r5 cell viability by MTT assay, showing cell growth, was significantly altered by the treatments of above the dose of HLP at 0.25 mg/mL, and was excluded in further studies (Figure 1c). In subsequent experimental migration research, the concentration range was used to avoid the influence of cell viability on the observed parameters. As shown in Figure 1d, it is worth noting the TNF-α-induced increase in MMP-9 activity was significantly inhibited in the cells incubated with the combinations of TNF-α together with this dose range of HLP (between 0.01 and 0.10 mg/mL).

### 3.2. HLP Downregulated TNF-α-Increased Protein and mRNA Levels of MMPs

**Figure 2:**
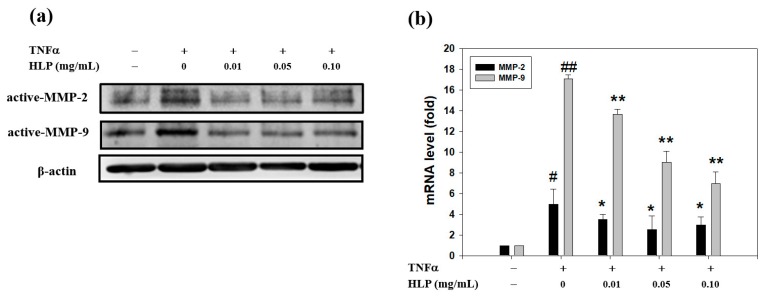
Effect of HLP on TNF-α-induced protein and mRNA levels of MMPs in VSMCs. A7r5 cells were treated with TNF-α (10 ng/mL) in the absence or presence of various concentrations (0, 0.01, 0.05, and 0.10 mg/mL) of HLP for 24 h. (**a**) Western blot analysis and (**b**) real-time quantitative RT-PCR of protein and mRNA levels of MMP-2 and MMP-9 in the treated cells. β-actin was served as an internal control of protein level. The quantitative data are presented as mean ± SD (*n* = 3) from three independent experiments. # *p* < 0.05, ## *p* < 0.01 compared with the control. * *p* < 0.05, ** *p* < 0.01 compared with the TNF-α group. +, added; −, non-added.

To understand further the downregulatory effects of HLP on the TNF-α-activated MMP-9, Western blotting was performed. As shown in Figure 2a, TNF-α elevated the protein levels of MMP-2 and MMP-9, and TNF-α together with the indicated concentrations of HLP (0.01, 0.05, and 0.10 mg/mL) caused a marked decreased level of MMP-9, but not MMP-2. The HLP-mediated decrease in the protein level of MMP-9 coincided well with its mRNA level, as evidenced by quantitative RT-PCR results (Figure 2b), indicating that HLP might downregulate the expression of MMP-9 majorly, but that of MMP-2 partially, at the transcriptional level.

### 3.3. HLP Inhibits TNF-α-Induced Akt/AP-1 Signaling

**Figure 3:**
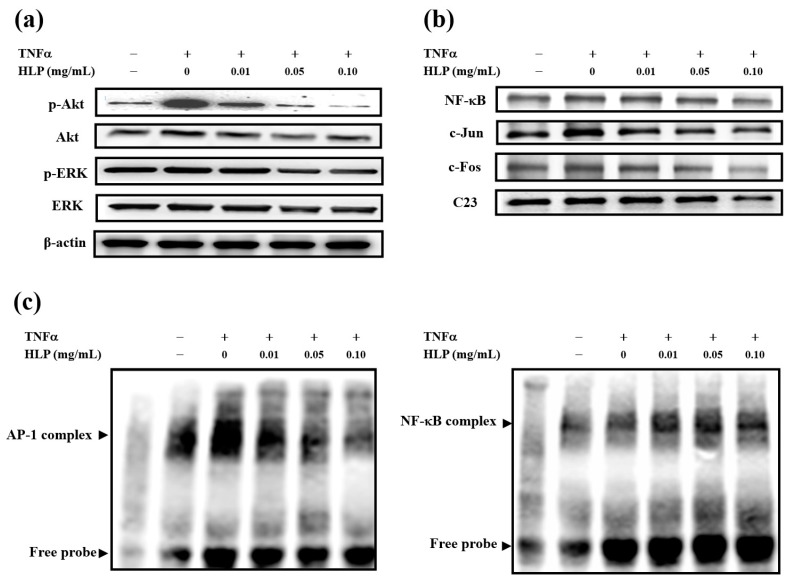
Effect of HLP on TNF-α-induced Akt/AP-1 signaling in VSMCs. A7r5 cells were treated with TNF-α (10 ng/mL) in the absence or presence of various concentrations (0, 0.01, 0.05 and 0.10 mg/mL) of HLP for 24 h; (**a**) the cytoplasmic fraction was analyzed for the expressions of p-Akt, Akt (protein kinase PKB, also known as Akt), p-ERK, and ERK (extracellular signal-regulated kinase), and (**b**) the nuclear fraction was analyzed for the expressions of NF-κB, c-Jun, and c-Fos, two components of activator protein-1 (AP-1). These protein levels were determined by Western blotting. β-actin and C23 served as a cytoplasmic and nuclear internal control, respectively. (**c**) The nuclear extracts were analyzed for AP-1 (left panel) and NF-κB (right panel) DNA-binding activities using biotin-labeled AP-1 and NF-κB specific oligonucleotide by electrophoretic mobility shift assay (EMSA). Lane 1 represents nuclear extracts incubated with unlabeled oligonucleotide (free probe) to confirm the specificity of binding. Results are representative of at least three independent experiments. +, added; −, non-added.

MAPK and Akt have been shown to be involved in MMP-9 induction in various tumor types and migratory cell phenotypes [5,23]. To examine whether the activities of these protein kinases are downregulated by HLP, we analyzed their phosphorylation in A7r5 cells after being exposed to 10 ng/mL of TNF-α in the presence or absence of HLP at the indicated concentrations for 24 h. Immunoblot analysis with anti-phospho-specific antibodies was then performed. As shown in Figure 3a, the TNF-α-induced phosphorylated level of Akt was tremendously reduced by HLP in a concentration-dependent manner, whereas that of ERK was little affected. MMP-9 promoter was shown to have several transcription-factor-binding motifs, including binding sites for AP-1 and NF-κB [23], indicating that the AP-1 and NF-κB signal pathway may play a key role in the regulation of MMP-9 expression. Therefore, whether HLP could interfere the translocation of AP-1 or NF-κB into the nucleus in TNF-α-stimulated VSMC by immunoblotting analysis of the nucleus extracts prepared from the treated cells was then tested. The data in Figure 3b demonstrate that stimulation with 10 ng/mL of TNF-α for 24 h induced significantly the nuclear levels of c-Jun, c-Fos, and NF-κB (p65), compared to that of the control group. After exposure to TNF-α for 24 h, HLP treatments inhibited nuclear levels of c-Jun and c-Fos, components of transcription factor AP-1, in a dose-dependent manner, with the higher concentrations (0.10 mg/mL) being more effective. In contrast, there was no noticeable change in the translocation of nuclear NF-κB in the same condition of HLP treatments. Furthermore, to confirm that HLP could affect the DNA-binding activities of the translocated AP-1 and NF-κB in the TNF-α model VSMC, EMSA was carried out. The nuclear extracts of the above-treated cells were incubated with a DNA probe specific for AP-1, and the binding was analyzed by mobility shift (Figure 3c). A decrease in the DNA binding activity of AP-1 (left panel), but not NF-κB (right panel), was presented in the cells treated with TNF-α in the presence of HLP at various concentrations for 24 h.

### 3.4. HLP Inhibits TNF-α-Induced Abnormal VSMC Migration

**Figure 4:**
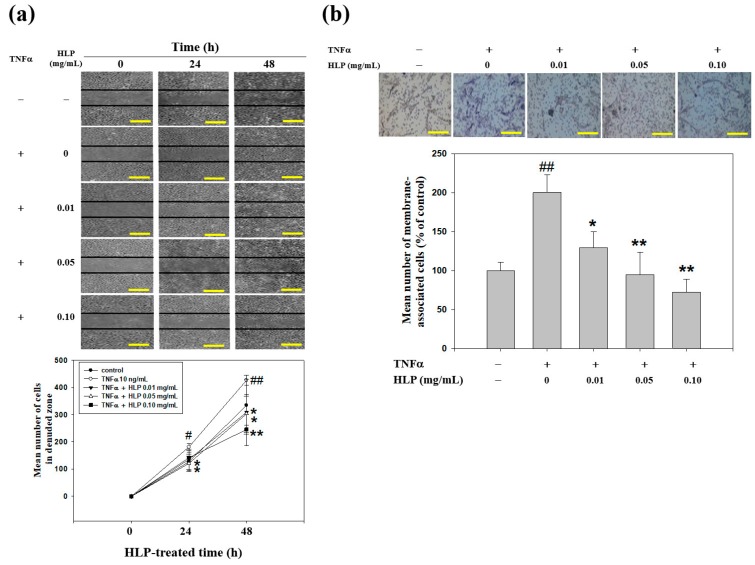
Effect of HLP on TNF-α-induced A7r5 cell motility and invasion. (**a**) Monolayers of A7r5 cells treated with TNF-α (10 ng/mL) in the absence or presence of various concentrations (0, 0.01, 0.05 and 0.10 mg/mL) of HLP were scraped and the number of cells in the denuded zone was photographed and quantified after indicated times (0, 24, and 48 h). Quantitative assessment of the mean number of cells in the denuded zone was presented as mean ± SD (*n* = 3) from three independent experiments. (**b**) A7r5 cells were treated with TNF-α in the absence or presence of various concentrations of HLP for 24 h. Invasion assay was performed using Boyden chamber. Representative photomicrographs of the membrane-associated cells were assayed by Giemsa stain. The purple parts indicate the membrane-associated cells. “% of control” denotes the mean number of cells in the membrane expressed as a proportion of that control group. Images were taken at 200× magnification; scale bar, 30 μm. The quantitative data are presented as mean ± SD (*n* = 3) from three independent experiments. # *p* < 0.05, ## *p* < 0.01 compared with the control. * *p* < 0.05, ** *p* < 0.01 compared with the TNF-α group. +, added; −, non-added.

To evaluate whether HLP reversed A7r5 cells from the TNF-α stimulation, a set of well-established and classical methods, wound-healing and Boyden chamber assays, was used to determine VSMC migration. The effect of HLP on abnormal VSMC migration was analyzed by wound-healing assay, in which A7r5 cells were induced to migrate by physical wounding of cells plated on fibronectin-precoated 6-well plates. Under light microscopy, an apparent and gradual increase of cells in the denude zone was observed at the cells exposed to TNF-α more than control for 24 and 48 h (Figure 4a). A7r5 cells treated with TNF-α, together with the indicated doses of HLP, showed a reduced capacity to heal the wounded area, compared to the TNF-α alone. The quantitative results demonstrate that HLP could dose- and time-dependently inhibit TNF-α-stimulated VSMC migration. Subsequently, the effect of HLP on VSMC invasion was examined by a Boyden chamber coated with Matrigel under light microscopy. After a 24-h incubation period, TNF-α promoted a marked increase in the amount of cell invasion. The results further show that the number of cells invaded to the lower chamber was significantly reduced by HLP treatments. The data in Figure 4b indicate that such decrease was dose-dependent, with a 70% decrease when the TNF-α model cells were treated with HLP at 0.01 mg/mL. Therefore, it is possible that the anti-VSMC migratory/invasive effect of HLP was conducted by inactivating Akt/AP-1, subsequently leading to a reduction in MMP-9 expression and activation in TNF-α stimulation.

### 3.5. HLP Inhibits TNF-α-Induced Abnormal VSMC Proliferation

**Figure 5:**
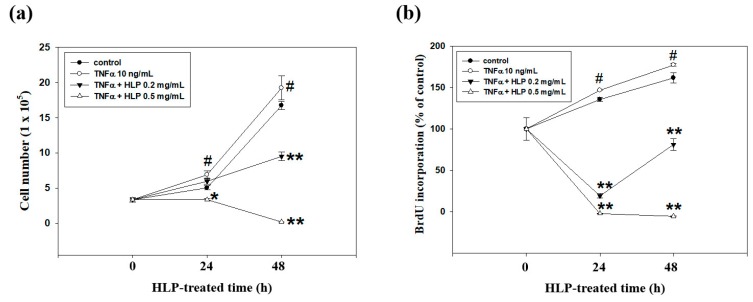

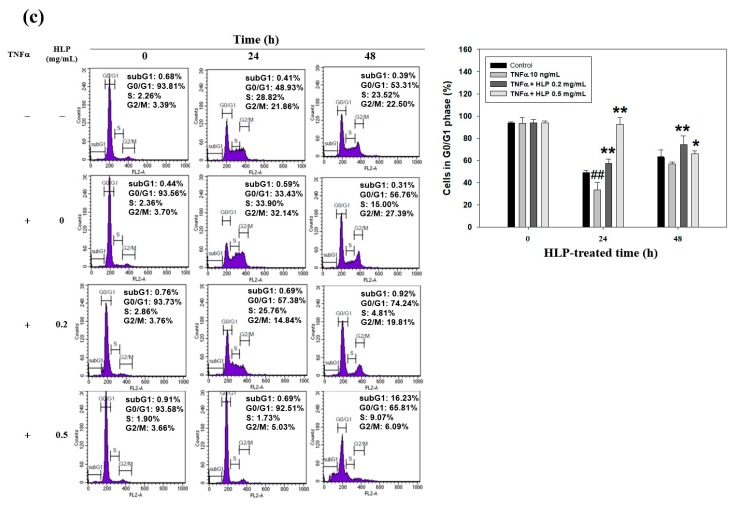
Effect of HLP on TNF-α-treated A7r5 cell growth curve, DNA synthesis, and cell cycle progression. A7r5 cells were treated with TNF-α (10 ng/mL) in the absence or presence of various concentrations (0, 0.2 and 0.5 mg/mL) of HLP for indicated time (0, 24 and 48 h). (**a**) The cell growth curve was evaluated using the Corning Cell Counter. (**b**) DNA synthesis was assayed by BrdU assay. (**c**) Cell cycle distribution was detected by flow cytometery. Quantitative assessment of the percentage of the cells in the cell cycle distribution (subG1, G0/G1, S, and G2/M phase) was indicated by PI dye. The proportion of cells in G0/G1 phase was quantitatively presented as mean ± SD (*n* = 3) of three independent experiments ± SD. # *p* < 0.05, ## *p* < 0.01 compared with the control. * *p* < 0.05, ** *p* < 0.01 compared with the TNF-α group. +, added; −, non-added.

In the following experiment, the cytotoxic effect of HLP at dosages above 0.10 mg/mL and TNF-α (10 ng/mL) was also detected using cell growth curve analysis. As shown in Figure 5a, the TNF-α-induced proliferation of A7r5 cells under the uses of TNF-α and HLP at 0.2 and 0.5 mg/mL was significantly lower than that under TNF-α alone. Importantly, the cell growth curve confirmed the anti-proliferative effect was more pronounced when HLP at the doses of > 0.10 mg/mL were used in the TNF-α-exposed cells. We then investigated whether the HLP effect against TNF-α was attributed by induction of cell death or/and inhibition DNA synthesis. For this purpose, the level of DNA synthesis through BrdU incorporation in the treated cells grown under low-serum conditions was measured. As shown in Figure 5b, TNF-α caused an increase in BrdU incorporation, and TNF-α together with higher doses of HLP had a marked decreased level in BrdU incorporation.

To further hypothesize that HLP may be involved in the VSMC cell death, flow cytometry was used to examine whether the number of hypodiploid cells (apoptotic cells), which are stained less intensely with PI dye, can be unequivocally detected in the subG1 phase (left panel, Figure 5c). When A7r5 cells were treated with TNF-α at 10 ng/mL in the presence of HLP at 0.2 mg/mL for 24 h, it was not observed that an apparent accumulation of cells in the subG1 phase. Here, the cell cycle distribution of TNF-α-treated VSMC affected by HLP was also evaluated. The 24-h TNF-α-stimulated cells showed a marked increase in S phase with fewer cells in G0/G1 phase, after TNF-α alone compared with control. When compared with the TNF-α alone group, the combination group had fewer cells in S phase and more cells in G0/G1 phase, indicating the 24-h HLP treatments could significantly lead to cell cycle block at G0/G1 phase in a dose-dependent manner (right panel, Figure 5c). In addition, when the cells were exposed to 0.5 mg/mL of HLP, a concomitant time-dependent slight and significant increase in apoptotic rates, compared to the TNF-α-treated group, was observed. Since the combination of HLP (0.2 mg/mL) and TNF-α (10 ng/mL) has the best antagonistic action of cell cycle regulation, the doses of combination were selected for further mechanistic studies of anti-VSMC proliferation, especially in G0/G1 arrest.

### 3.6. HLP Induces Cell Cycle Arrest in the Present of TNF-α

**Figure 6:**
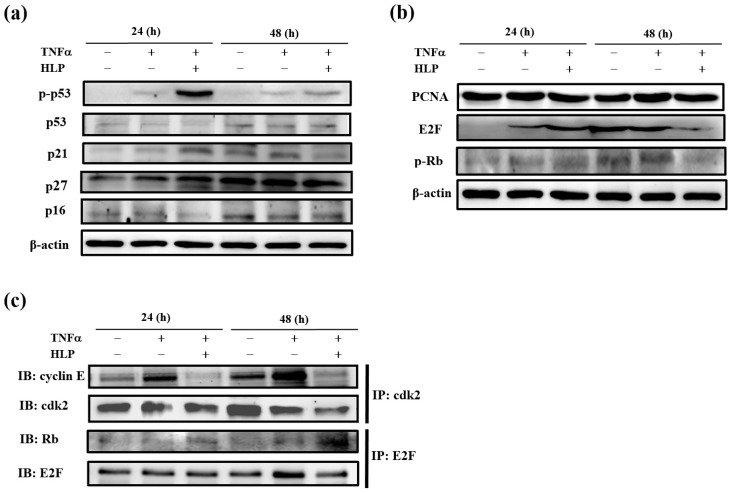
Effect of HLP on TNF-α-regulated the expressions of cell cycle regulatory proteins in VSMCs. A7r5 cells were treated with TNF-α (10 ng/mL) in the absence or presence of 0.2 mg/mL of HLP for 24 and 48 h. The protein levels of cdi, including p-p53, p53, p21, p27, and p16 (**a**), anti-proliferating cell nuclear antigen (PCNA), E2F, and p-Rb (**b**) were determined by Western blotting. β-actin was served as an internal control. (**c**) The expressions of cyclin E/cdk2 and Rb/E2F complexes were further analyzed. The cell extracts were immunoprecipitated (IP) with cdk2 or E2F. The precipitated complexes were examined for immunoblotting (IB) using anti-cyclin E or Rb antibody. Results are representative of at least three independent experiments. +, added; −, non-added.

To investigate further the mechanism of the effect HLP on cell cycle arrest at G0/G1 phase, A7r5 cells treated with TNF-α (10 ng/mL) in the presence or absence of HLP (0.2 mg/mL) for 24 and 48 h were subjected to immunoblot analysis. We first analyzed the expressions of phospho-p53 (p-p53), p53, and cki, including p16, p21, and p27. Among them, p-p53, p21, and p27 levels were significantly induced by a 24-h HLP treatment (Figure 6a). To further investigate whether the inhibitory effect of HLP on TNF-α occurred because it blocked A7r5 cell cycle progression, the changes in protein levels of PCNA, E2F, and p-Rb, regulators of cell cycle G0/G1 arrest, were also studied (Figure 6b). Stimulation with TNF-α at 10 ng/mL for not only 24 h, but also 48 h, promoted time-dependently the expressions of PCNA, E2F, and p-Rb, compared to the receptive control group. After exposure to TNF-α for 48 h, the HLP treatment significantly inhibited three expressions (Figure 6b).

Using immunoprecipitation, we confirmed that the addition of TNF-α upregulated the formation of cyclin E/cdk2 complex without noticeable change in cyclin D/cdk4 complex (data not shown) in A7r5 cells at 24 to 48 h, but HLP reversed the increases (line 1, Figure 6c). Moreover, there was a more significant increase in expression of Rb/E2F complex in the TNF-α combined with HLP treatments group (Figure 6c). As shown in Figure 6c (line 3), an increase in Rb/E2F complex was correlated with a decrease in p-Rb at 48 h of cell cycle (line 3, Figure 6b). The HLP-increased expression of Rb/E2F complex prevented the release of E2F transcription factor, and then reduced the transcription of the genes required for the cell cycle progression (Figure 6b,c). These data show that HLP regulated the association of cyclin E/cdk2, and Rb/E2F, inducing the cell cycle arrest at G0/G1 phase of A7r5 cells in the presence of TNF-α.

### 3.7. HLP Reduced Atherosclerotic Lesions, and the Abnormal Migration and Proliferation of VSMC in a Rabbit Model

**Figure 7:**
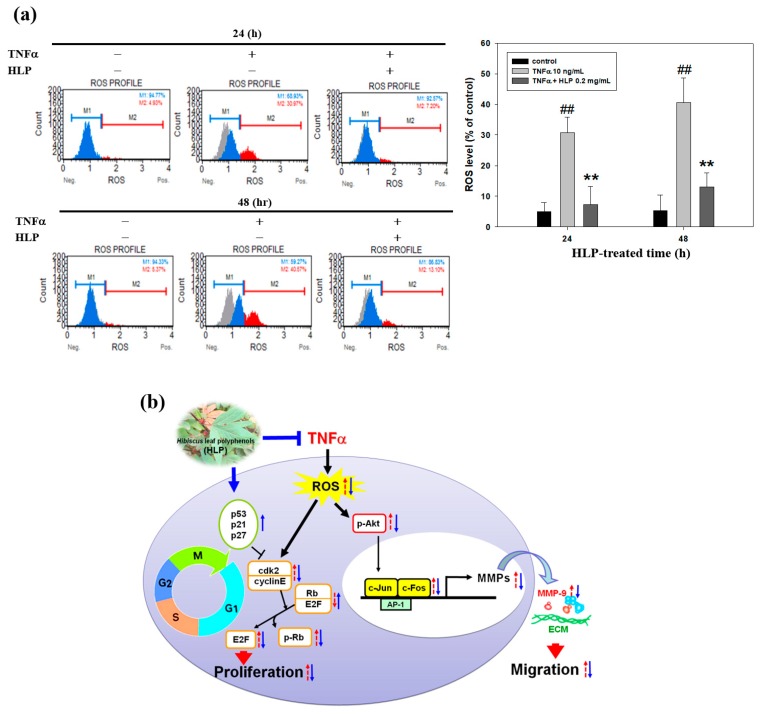
Effect of HLP on TNF-α-induced ROS production in VSMC. (**a**) A7r5 cells were treated with TNF-α (10 ng/mL) in the absence or presence of 0.2 mg/mL of HLP for 24 and 48 h. The treated cells were then labeled with fluorescent probe, dichlorofluorescin diacetate (DCFH-DA), and reactive oxygen species (ROS) production was measured using Muse™ Cell Analyzer. M1: DCF-negative cells. M2: DCF-positive cells. The results are presented as mean ± SD (*n* = 3) from three independent experiments. ## *p* < 0.01 compared with the control. ** *p* < 0.01 compared with the TNF-α group. +, added; −, non-added. (**b**) Schematic representation of TNF-α-antagonist potential of HLP on VSMCs. TNF-α induces intracellular ROS production, leading cell migration and proliferation through Akt/AP-1/MMP-9 signaling and cyclin E/cdk2-mediated Rb phosphorylation in A7r5 cells. HLP functions against TNF-α via downregulation of Akt/MMP-9 and upregulation of p53 signals that subsequently inhibit VSMC migration and proliferation. Red arrows represent the changes in response to TNF-α stimulation; blue arrows represent changes in TNF-α-exposed VSMCs receiving HLP intervention.

Oxidant stress is a major cause of VSMC dysfunction and inflammation through various pathways [6,7]. To investigate the antioxidant action of HLP resulting from VSMC dysfunction, the ROS generation (DCF fluorescence) following the HLP treatments in the TNF-α-stimulated cells was examined (left panel, Figure 7a). The results showed that TNF-α significantly increased the fluorescence of intracellular ROS generation at not only 24 h, but also 48 h, whereas HLP at 0.2 mg/mL inhibited production of intracellular ROS (right panel, Figure 7a), implicating its antioxidant effects. In the same condition, the inhibitory effect of HLP on the amount of hydrogen peroxide (H_2_O_2_), the major form of ROS, was similar to the result of ROS production upon TNF-α stimulation (Figure S2). Collectively, these results suggest that cyclin E/cdk2-dependent Rb phosphorylation and Akt/AP-1/MMP-9 signaling pathway mediated the in vitro action of HLP against to TNF-α-induced ROS production, controlling the balance of VSMC proliferation and migration (Figure 7b).

### 3.8. HLP Reduced Atherosclerotic Lesions and the Abnormal Migration and Proliferation of VSMC in a Rabbit Model

**Figure 8:**
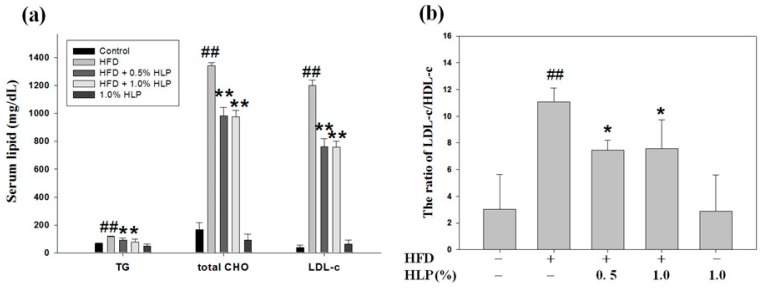

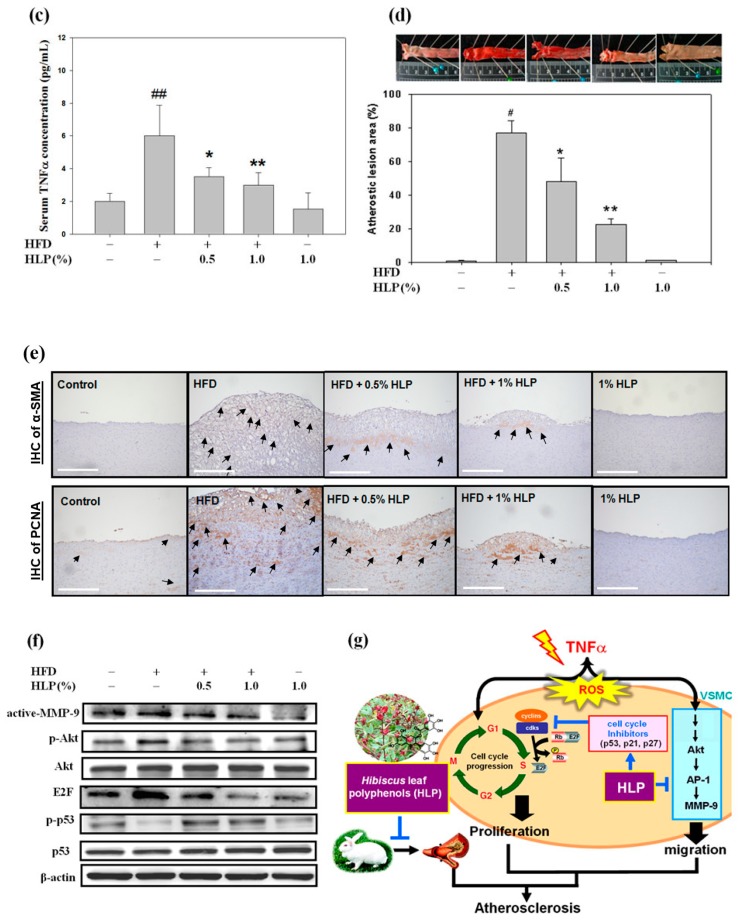
Effect of HLP on atherosclerotic lesions, and VSMC migration and proliferation in vivo. Among five groups, New Zealand white rabbits fed on a high-fat diet (HFD) were divided into three groups. At the same time, two of the groups were orally treated with HLP at a dose of 0.5% or 1.0%. These rabbits were sacrificed after 25 weeks. The serum levels of triglycerides (TG), total cholesterol (CHO), low-density lipoprotein cholesterol (LDL-c) (**a**), ratio of LDL-c/ high-density lipoprotein cholesterol (HDL-c) (**b**), and TNF-α (**c**) were determined by ELISA assays. The results are presented as mean ± SD from one independent experiment. # *p* < 0.05, ## *p* < 0.01 compared with the control. * *p* < 0.05, ** *p* < 0.01 compared with the HFD group. The aortic arches were collected for (**d**) oil red O stain, (**e**) immunohistochemistry (IHC) staining of α-SMA (upper panel), PCNA (lower panel). Images were taken at 400× magnification; scale bar, 50 μm. (**f**) Western blot analysis of MMP-9, p-Akt, Akt, E2F, p-p53, and p53 protein expressions was carried out with the tissue extracts from them. β-actin was served as an internal control. Results are representative of at least three independent experiments. +, added; −, non-added. (**g**) Overview of pathways for the proposed mechanism of HLP-induced inhibition of atherosclerosis in rabbits and VSMC migration/proliferation.

Because abnormal VSMC migration and proliferation contribute significantly in the pathogenesis of cardiovascular diseases, improvements in VSMC dysfunction will prevent the development of atherosclerosis [24]. For the clinical use of HLP for atherosclerosis, we investigated the HLP effect against VSMC dysfunction, using an atherosclerotic rabbit model. As shown in Figure 8a,b, HLP can significantly reduce the elevation of the concentrations of serum triglycerides (TG), total cholesterol (CHO), and LDL cholesterol (LDL-c) (Figure 8a), and the ratio of LDL-c and high-density lipoprotein cholesterol (HDL-c) (Figure 8b) enhanced by a HFD treatment. Past reports have shown that the decrease of LDL-c/HDL-c ratio, not just the LDL-c level alone, is of a lot of importance for reducing the atheroma burden [24,25]. In addition, the serum level of TNF-α was also significantly reduced after HFD-fed rabbits were treated with HLP (Figure 8c), confirming that HLP has a TNF-α antagonistic effect. Our study showed, in addition to possessing benefits to serum lipids, HLP can effectively decrease serum LDL/HDL ratio and TNF-α level, thus improving atherosclerosis.

To evaluate the in vivo atheroprotective effect of HLP against the extent of atherosclerosis in the aorta, the area of fatty region in the atherosclerotic lesions was analyzed using oil Red-O staining. The data in Figure 8d reveal that the subintimal lipid deposition in the HFD-treated rabbits was improved after HLP treatment. In addition, IHC staining indicated the expressions of α-SMA (upper panel) and PCNA (lower panel), served receptively as markers of VSMC migration and proliferation, were showed in the intima of atherosclerotic lesions from aortic roots of the rabbits treated with HFD (Figure 8e). As shown in Figure 8e, VSMC dysfunction was significantly observed in the atherosclerotic lesions in the HFD-treated rabbits, but very few expressions of α-SMA and PCNA in the HFD plus HLP-fed rabbits, which was consistent with the HLP-reduced the cell migration and proliferation in TNF-α-treated A7r5 cells in vitro (Figure 4 and Figure 5). In the treatment process, HLP administration did not have any adverse effects on body weight or liver and renal function, compared to those of control (data not shown). Additionally, Western blotting of tissue extracts in the aortic arch showed the expressions of active-MMP-9, p-Akt, and E2F were markedly decreased, but the phosphorylation of p53 was increased in the group of HFD plus HLP, when compared with HLP or HFD-fed groups (Figure 8f). These results indicate that HLP can significantly improve VSMC dysfunction of HFD-treated rabbits by inhibiting cell migratory and proliferative signal pathways in vivo, as well as in vitro (Figure 8g).

## 4. Discussion

*H. sabdariffa* Linne (*Malvaceae*), an attractive plant believed to be native to African countries, is cultivated in both Southern and Eastern Taiwan [26]. The calyces of the plant are typically used in foods and beverages, such as jam, jellies, and teas [15,26]. Previous studies have shown that various extracts of calyces of *H. sabdariffa* L., including *H. sabdariffa* aqueous extracts (HSEs), *H. sabdariffa* anthocyanins (HAs), and its polyphenol-rich extracts (HPEs), have been reported to exhibit a wide variety of activities against hypertension, inflammation, liver disorders, diabetes, cancer, atherosclerosis, and other metabolic syndromes [26]. While the focus has been on the calyx, the leaves of this plant are also consumed as a leafy vegetable in many countries [15]. The *Hibiscus* leaf has been also reported to exert many biologic effects, including antioxidant [13,15], anti-hyperlipidemic [13,14], anti-cancer [17,18], anti-atherosclerotic [11,16], and anti-inflammatory [15] activities, as shown in Table S1. Our recent studies have indicated that HLP, a methanol extract of *Hibiscus* leaf, is rich in polyphenols [16], including ECG and other polyphenols (Cat, EA, Que, and FA; Table S2). In the literature, ECG-enriched HLP exhibited to inhibit ox-LDL uptake and lipid-laden foam cell formation, promoted cholesterol efflux [16], and reduced ox-LDL-mediated endothelial cell injury and apoptosis [11], so HLP was expected to have potential as an anti-atherogenic agent. ECG, one of the major tea catechins, plays an important protective role in the cardiovascular system, and has been reported to possess anti-atherogenic properties in in vitro and in vivo studies [27]. It has been shown that the anti-atherosclerotic effect of Cat is associated with their antioxidant, anti-hypertensive, hypolipidaemic, and anti-mutagenic effects [28]. These Cat have been indicated to suppress the LDL oxidation and the foam cell formation in in vivo atherosclerotic lesions [27], and MMP-2 activities in cell culture supernatant of pulmonary VSMC [29]. Previous studies have indicated that EA, a polyphenolic compound present in berries, scavenged free radicals and improved lipid peroxidation [30]. According to the past and present works, the findings cooperatively show the anti-atherosclerotic activities of HLP may be contributed by their biological properties of these polyphenolic components.

In the comparison of components between both extracts, polyphenolic extracts from flowers (HPE) and leaves (HLP) of *H. sabdariffa* L., the total flavonoid content of HPE and HLP was estimated to near to 20% and 75%, respectively, via Jia method [16,22]. The results show the polyphenolic extract of *Hibiscus* leaves exhibited an about 3.8-fold content of flavonoids compared to that of its flowers. In addition to the above, HLP seems to possess stronger protective properties from VSMC dysfunction than HPE [22], including the inhibitory effects on MMP expression and cell migration (Figure 2 and Figure 4). Huang et al. demonstrated that HPE at the doses of 0.01 and 0.10 mg/mL reduced high glucose-stimulated cell migration about 30% and 80%, respectively, by measuring the wound-healing assay [22]. In this study, above the dose of HLP at 0.01 mg/mL could completely reverse the TNF-α-increased proportion of cell migration (Figure 4a). These data suggest that HLP could exert the anti-migratory effect at lower doses than HPE.

Atherosclerosis is a multistep and chronic inflammatory process that involves interactions between various soluble mediators, endothelial cells, monocytes, and VSMCs. Monocyte-derived growth factors and cytokines further affect the vascular wall by stimulating VSMC migration and proliferation [31]. The inflammatory cytokine TNF-α has been shown to play a vital role in the disruption of the vascular circulation, and its increased expression induces the production of ROS, resulting in endothelial cell injury and VSMC dysfunction [32]. The blockade of TNF-α has been demonstrated to improve cardiovascular morbidity and mortality in chronic inflammatory disease [33]. The model of TNF-α-stimulated VSMCs has been applied to mimic the VSMC dysfunction during atherosclerotic development [31]. Therefore, the atheroprotective effects of HLP were investigated in a model of VSMCs exposed to TNF-α in vitro. The extract, at a concentration in a range of 0.01–0.10 mg/mL, possessed inhibitory effects on VSMC migration, as evidenced by the results of the decreased activities and expressions of MMPs, the levels of key migratory proteins, and the wound-healing and invasive abilities of VSMCs (Figure 1d and Figure 2, Figure 3 and Figure 4). The effect of the higher concentrations (>0.10 mg/mL) showed that it repressed cell growth and DNA synthesis, as well as enhanced cell apoptosis (Figure 5). Similarly, Won et al. reported that the mechanisms of catechins action against cardiovascular diseases include the inhibition of VSMC proliferation [34]. Further studies have also indicated that many polyphenols prevented atherosclerosis through inhibiting proliferation and/or inducing apoptosis of VSMC [35]. The findings of this study reveal, for the first time, the protective effects of HLP on this model, examined in each test, provide bifunctional results of HLP. Therefore, it is convincing that HLP could potentially be used in the treatment of atherosclerosis.

As mentioned above, the migration of VSMCs from medial to intima contributes to the formation of atherosclerosis [2]. MMPs, a family of proteinases, promote ECM degradation, which, in turn, facilitates cell migration. Activated Akt induced AP-1, which is required for the production of MMPs [36]. However, upon TNF-α stimulation, the mechanism(s) mediating MMP activation and the MMP-regulated downstream signals have yet to be clarified. TNF-α promoted VSMC chemotaxis via Akt and MAPK activation, as reported by Chan et al. [31]. The Akt antagonist PTEN was also shown to be involved in VSMC migration [37]. Consistent with previous reports, this study confirmed that it is therefore possible that HLP inhibits TNF-α-stimulated MMP-9 activation by downregulating Akt/AP-1 pathway, and subsequently prevents VSMC migration. Furthermore, previous studies have indicated that oxidative stress modulated by ROS promotes VSMC dysfunction developing atherosclerosis and also induces MMP-mediated ECM remodeling and cell cycle progression [6,7,38]. In this study, TNF-α increased the production of intracellular ROS, especially of the H_2_O_2_ level, and this might influence the overall signaling pathways of VSMC migration/proliferation. In this regard, HLP attenuated ROS generation against TNF-α stimulation (Figure 7), and it might contribute to MMP inhibition and cell cycle regulation by HLP. Further investigations are needed to clarify this issue. Consistent with previous reports and our past results, as shown in Table S4, this study confirmed that HLP possesses strong antioxidant ability in inhibiting the VSMC dysfunction and atherosclerotic development.

Next, to study the mechanism(s) of HLP-inhibited VSMC proliferation, the regulation of cell cycle arrest was examined. VSMCs begin to divide in response to mitogens and enter the S phase upon vascular injury [9]. Cdks, working in conjunction with their activating subunits (cyclins), provide the driving force for cell cycle transitions [34]. The kinase activities of these cyclin/cdk complexes are regulated by cdi, including Ink4 proteins (p16, p18, and p19) and Cip/Kip proteins (p21, p27, and p57), and their upstream factor, the gatekeeper of mammalian cell cycle, p53 [10,39]. As expected, HLP treatments induced a G0/G1 phase growth arrest by inducing the cki-mediated cell cycle regulation (Figure 6). In addition, PCNA expression is increased in unstable atherosclerotic plaque [40]; moreover, PCNA is highly expressed in human VSMCs [41]. Consistent with previous reports, our studies showed that TNF-α significantly reduced A7r5 cell growth and increased PCNA expression (Figure 6b). Further data demonstrate a marked and dose-dependent reduction in neointimal expression of PCNA after fed with HLP in HFD-treated rabbits (Figure 8e), indicating that HLP ameliorates atherosclerosis by reducing PCNA expression, both in vitro and in vivo.

## 5. Conclusions

In conclusion, the findings of this study are schematically presented in Figure 8g. TNF-α generally induces ROS production, which influences Akt/AP-1/MMP-9 signaling and cyclin E/cdk2-mediated phosphorylation of Rb, leading to participation in abnormal VSMC migration and proliferation. Conversely, HLP is speculated to play a role in TNF-α antagonist, which contributes to inhibiting ROS generation and VSMC dysfunction. Because our in vitro data demonstrate that HLP inhibited TNFα-induced VSMC migration and proliferation, in vivo results indicate that HLP can effectively improve HFD-promoted atherosclerosis in rabbits via decreasing TNF-α secretion to confirm the anti-atherosclerotic effect of HLP (Figure 8a–d). As shown in Figure 8, HLP can pleiotropically inhibit rabbit atherosclerosis by reducing serum lipid levels, inflammation, atheroscletic lesion, and VSMC migration/proliferation via downregulation of Akt/MMP-9 and upregulation of p53 signals. Taken together, our findings indicate the anti-TNF-α effects of HLP on VSMC could likely contribute to its protection against atherosclerosis and other cardiovascular disorders.

## Supplementary Materials

The following are available online at https://www.mdpi.com/2076-3921/8/12/620/s1: Table S1. Biological activities of *Hibiscus* leaf in previous studies; Table S2. Polyphenolic compound content (in %) in methanol extracts of *Hibiscus* leaf; Table S3. Sequences of primers used in RT-PCR; Table S4. Antioxidant capacity of HLP in standard antioxidant evaluation; Figure S1. Effect of HLP alone on cell viability in VSMCs; Figure S2. Effect of HLP on TNF-α-induced H_2_O_2_ production in VSMC.
