# Supplementary material for: Anti-Atherosclerotic Effect of Hibiscus Leaf Polyphenols against Tumor Necrosis Factor-alpha-Induced Abnormal Vascular Smooth Muscle Cell Migration and Proliferation
Source: Antioxidants (Basel). 2019 Dec 5;8(12):620. doi: 10.3390/antiox8120620 (PMC6943519; doi:10.3390/antiox8120620)
Supplement: Supplementary file 1 [file antioxidants-08-00620-s001.pdf]

Supplementary Material

Chemicals

Polyphenols standards [gallic acid (GA), rutin (Rut), protocatechuic acid (PCA), catechin (Cat), (−)-epicatechin gallate (ECG), ellagic acid (EA), -coumaric acid (CA), ferulic acid (FA), quercetin (Que), and naringenin (Nar)], TNF , dimethyl sulfoxide (DMSO), 3-(4, 5-dimethylthiazol-zyl)-2,5-diphenyltetrazolium bromide (MTT), sodium dodecyl sulfate (SDS), gelatin, Triton X-100, Tris-HCl, Nonidet P-40, β-mercaptoethanol, secondary antibodies, Matrigel, Giemsa, propidium iodide (PI), RNase A, cholesterol, and lard oil were purchased from Sigma-Aldrich (St Louis, MO, USA). Dulbecco's modified Eagle's medium (DMEM), fetal bovine serum, penicillin–streptomycin mixed antibiotics, glutamine, sodium bicarbonate, phosphate-buffered saline (PBS), and trypsin-EDTA were from Hyclone (Logan, UT, USA). TRIzol reagent was obtained from Invitrogen (Life Technologies, Carlsbad, CA, USA), and protease inhibitor cocktail and nitrocellulose membranes were purchased from Bio-Rad Labs. (Hercules, CA, USA). Polyclonal antibodies against MMP-2, MMP-9, p-Akt, Akt, p-ERK, ERK, c-Jun, c-Fos, NF- B, p-p53, p53, p21, p27, p16, PCNA, E2F, p-Rb, -actin, C23, cdk2, cyclinE, and α-SMA were from Santa Cruz Biotech (CA, USA). The enhanced chemiluminescence (ECL) reagent was purchased from Amersham (Arlington Heights, IL).

Supplementary Data

Table S1. Biological activities of *Hibiscus* leaf in previous studies.

Biological Activity	Experimental Model	Reference
Hypoglycemic	Glucose- and streptozotocin-induced hyperglycemic rats	Sachdewa et al., 2001 [12]
Hypolipidemic	Cholesterol-induced hyperlipidemic rats	Ochani and D'Mello, 2009 [13]; Gosain et al., 2010 [14]
Antioxidant	Cholesterol-induced hyperlipidemic rats cell-free system	Ochani and D'Mello, 2009 [13]; Zhen et al., 2016 [15]
Anti-atherogenic	Oxidized LDL-induced murine macrophages and human endothelial cells cholesterol-induced hyperlipidemic rats	Chen et al., 2017 [11]; Ochani and D'Mello, 2009 [13]; Gosain et al., 2010 [14]; Chen et al., 2013 [16]
Anti-cancer	Human prostate cancer cells	Lin et al., 2012 [17]; Chiu et al., 2015 [18]
Anti-inflammatory	Lipopolysaccharide-induced murine macrophages	Zhen et al., 2016 [15]

Table S2. Polyphenolic compound content (in %) in methanol extracts of *Hibiscus* leaf.

Compound [a]	Abb [b]	Rt [c] (min)	Content [d] (%)	Statistics
Catechin	Cat	9.3	7.4 ± 2.6	$p < 0.01$
(−)-Epicatechin gallate	ECG	11.2	16.5 ± 5.6	$p < 0.01$
Ellagic acid	EA	13.3	10.3 ± 3.4	$p < 0.01$
Ferulic acid	FA	15.2	0.7 ± 0.3	$p < 0.01$
Quercetin	Que	21.5	0.8 ± 0.4	$p < 0.01$
Total phenolic acid (Folin–Ciocalteu method)			34.5 ± 1.1	$p < 0.01$
Total flavonoid (Jia method)			65.2 ± 9.4	$p < 0.01$

[a] Polyhenolic compounds correspond to peaks as in HPLC chromatogram of 10 kinds of standard polyphenols, including gallic acid, protocatechuic acid, catechin, (−)-epicatechin gallate, ellagic acid, rutin, ϱ-coumaric acid, ferulic acid, quercetin, and naringenin. [b] Abbreviation. [c] Retention time. [d] The content of each polyphenol is expressed as percentage of the polyphenolic compounds in *Hibiscus* leaf polyphenol (HLP), quantified relative to standards, and represents the average of three independent experiments. Means in a row without a common letter differ, $p < 0.05$.

Table S3. Sequences of primers used in RT-PCR.

Target Gene	Primer	Nucleotide Sequence
MMP-2	Forward	5′-CTGACCCCCAGTCCTATCTGCC-3′
	Reverse	5′-TGTTGGGAACGCCTGACTTCAG-3′
MMP-9	Forward	5′-CTTTGACAGCGACAAGAAGTGG-3′
	Revers	5′-GGCACTGAGGAATGATCTAAGC-3′
-actin	Forward	5′-CTGGAACGGTGAAGGTGACA-3′
	Revers	5′-AAGGGACTTCCTGTAACAATGCA-3′

Table S4. Antioxidant capacity of HLP in standard antioxidant evaluation [a].

Antioxidant Assays	HLP Concentration (mg/mL)			
	0.01	0.05	0.1	0.2
DPPH scavenging effect (% of control) [b]	8.80 ± 0.24 *	11.83 ± 0.93 *	33.53 ± 0.87 **	44.53 ± 2.03 **
TBARS inhibition effect (% of ox-LDL) [c]	6.64 ± 5.18	85.68 ± 1.15 **	89.45 ± 1.52 **	90.15 ± 1.59 **
REM inhibition effect (% of ox-LDL) [d]	22.08 ± 5.66	36.73 ± 2.45 *	80.22 ± 8.10 *	87.75 ± 15.03 *
ApoB remaining effect (% of ox-LDL) [e]	3.67 ± 3.52	4.81 ± 3.06 *	20.74 ± 8.08 *	79.98 ± 28.54 **

[a] The results in our previous studies have been published and described by Chen et al. (2013) [14]. [b] 1,1-diphenyl-2-picrylhydrazyl (DPPH) was incubated with different concentrations of HLP (0–0.2 mg/mL) for 30 min. The result represents the average of three independent experiments ± standard deviation (SD). * $p < 0.05$, ** $p < 0.01$ compared with the control. [c–e] Low-density lipoprotein (LDL) was incubated with 10 M CuSO4 at 37 °C for 24 h in the presence or absence of different concentrations of HLP. After the incubation, the lipid peroxidation of LDL, assessed by measuring the thiobarbituric acid relative substances (TBARS), and relative electrophoretic mobility (REM) and apolipoprotein B (ApoB) fragmentation of the LDL were analyzed. The result represents the average of three independent experiments ± SD. * $p < 0.05$, ** $p < 0.01$ compared with the oxidized LDL (ox-LDL) group.

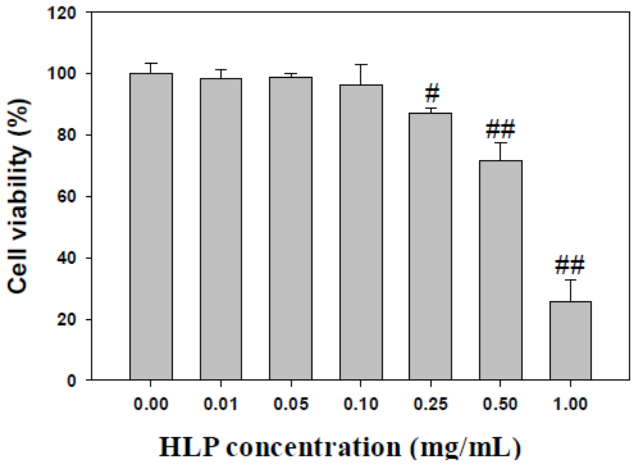

Figure S1. Effect of HLP alone on cell viability in vascular smooth muscle cells (VSMCs). A7r5 cells were treated with various concentrations (0–1.00 mg/mL) of HLP for 24 h. Cell viability was analyzed by MTT assay. The quantitative data are presented as mean ± SD (n = 3) from three independent experiments. # $p < 0.05$, ## $p < 0.01$ compared with the control.

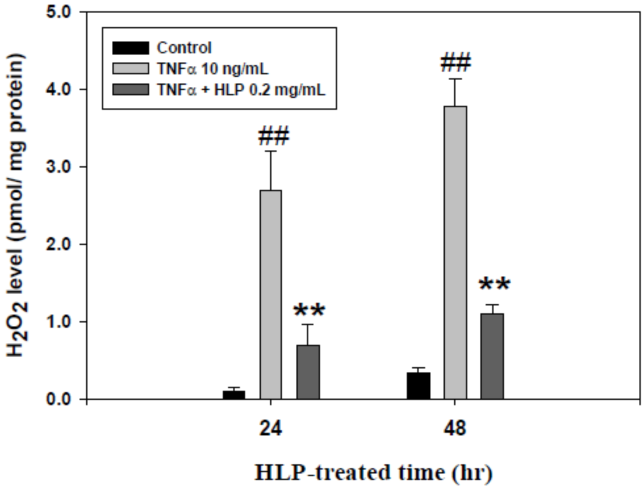

Figure S2. Effect of HLP on TNF -induced H_2O_2 production in VSMCs. A7r5 cells were treated with TNF (10 ng/mL) in the absence or presence of 0.2 mg/mL of HLP for 24 and 48 h. H_2O_2 production was measured by H_2O_2 assay. The results are presented as mean ± SD (n = 3) from three independent experiments. ## $p < 0.01$ compared with the control. ** $p < 0.01$ compared with the TNF group.
